# Recent Trends in the Labor Market for Older Workers and Their Impact on Retiree Well-Being

**DOI:** 10.1093/geroni/igaf122.1434

**Published:** 2025-12-31

**Authors:** Joseph Quinn, Kevin Cahill, Christine Bishop

## Abstract

The labor market for older workers continues to change, and these changes can have far-reaching impacts on the well-being of retirees. This symposium covers some of the key trends in the labor force participation of older workers, with an emphasis on gender and racial/ethnic differences and wealth outcomes later in life. The first paper explores how trends in labor force participation have differed historically for men and women, and how these gender differences appear to have stabilized since 2010. The second paper explores the impact of sandwich caregiving on labor force participation, and how these impacts differ by key subgroups. The third paper explores labor force participation in the unique context of Turkey, where the pension system has developed all in living memory. The fourth paper connects labor market experiences with retirement outcomes, by examining structural barriers to racial/ethnic equity in retirement. The fifth paper rounds out the symposium by exploring the pivotal relationships between cognitive decline, financial decision making, and the wealth outcomes of older households. The flexibility of the labor market has real benefits for workers generally and for older workers in particular. Indeed, the ubiquitousness of gradual retirement reflects how older workers customize their transitions out of the labor force. The flexibility of the labor market comes with clear trade-offs, however, as structural challenges have left some individuals behind. This symposium provides much-needed context on the labor market outcomes of older workers and how these outcomes impact the financial well-being of older individuals into their retirement years. Economics of Aging Interest Group Sponsored Symposium

